# Positive Association between Urinary Concentration of Phthalate Metabolites and Oxidation of DNA and Lipid in Adolescents and Young Adults

**DOI:** 10.1038/srep44318

**Published:** 2017-03-14

**Authors:** Chien-Yu Lin, Pau-Chung Chen, Chia-Jung Hsieh, Chao-Yu Chen, Anren Hu, Fung-Chang Sung, Hui-Ling Lee, Ta-Chen Su

**Affiliations:** 1 Department of Internal Medicine, En Chu Kong Hospital, New Taipei City 237, Taiwan; 2 School of Medicine, Fu Jen Catholic University, New Taipei City 242, Taiwan; 3 Institute of Occupational Medicine and Industrial Hygiene, College of Public Health, National Taiwan University, Taipei 100, Taiwan; 4 Department of Public Health, College of Public Health, National Taiwan University, Taipei 100, Taiwan; 5 Department of Environmental and Occupational Medicine, National Taiwan University College of Medicine and National Taiwan University Hospital, Taipei 100, Taiwan; 6 Department of Public Health, Tzu Chi University, Hualian County 970, Taiwan; 7 Department of Chemistry, Fu Jen Catholic University, New Taipei City 242, Taiwan; 8 Department of Laboratory Medicine and Biotechnology, Tzu Chi University, Hualian County 970, Taiwan; 9 Department of Health Services Administration, College of Public Health, China Medical University, Taichung 404, Taiwan; 10 Department of Internal Medicine and Cardiovascular Center, National Taiwan University Hospital, Taipei 100, Taiwan

## Abstract

Phthalate has been used worldwide in various products for years. Little is known about the association between phthalate exposure and biomarkers of oxidative stress in adolescents and young adults. Among 886 subjects recruited from a population-based cohort during 2006 to 2008, 751 subjects (12–30 years) with complete phthalate metabolites and oxidation stress measurement were enrolled in this study. Nine urine phthalate metabolites, 8-hydroxydeoxyguanosine (8-OHdG), and 8-iso prostaglandin F2α (8-isoPGF2α) were measured in urine to assess exposure and oxidative stress to DNA and lipid, respectively. Multiple linear regression analysis revealed that an ln-unit increase in mono-methyl phthalate (MMP) concentration in urine was positively associated with an increase in urine biomarkers of oxidative stress (in *μ*g/g; creatinine of 0.098 ± 0.028 in 8-OHdG; and 0.253 ± 0.051 in 8-isoPGF2α). There was no association between other eight phthalate metabolite concentrations and oxidative stress. In conclusion, a higher MMP concentration in urine was associated with an increase in markers of oxidative stress to DNA and lipid in this cohort of adolescents and young adults. Further studies are warranted to clarify the causal relationship between exposure to phthalate and oxidative stress.

Phthalates (diesters of 1 2-benzenedicarboxylic acid) are a group of chemicals that are widely used as plasticizers in various domestic and industrial products to increase the flexibility, durability, and transparency of plastics such as polyvinyl chloride and cellulose acetate. These chemicals are often used as plasticizers or solvents in food packaging, cosmetics, perfumes, nail polishes, flooring, and other industrial products. These chemicals can enter the human body through daily ingestion and inhalation^1^,^2^. After exposure, phthalates undergo a series of phase I hydrolysis and phase II conjugation reactions and are excreted in feces and urine^3^. Human are widely exposure to phthalates due to its metabolites are detected ubiquitously in urine samples in the US and elsewhere^4^. Although phthalates have relatively short half-lives (approximately 12 hours)^5^, continuous daily exposure leads to effects similar to those caused by persistent and bioaccumulative compounds^6^. In May 2011, the Taiwan Food and Drug Administration reported that di-(2-ethylhexyl) phthalate (DEHP) and di-iso-nonyl phthalate were illegally used to replace palm oil in food and drinks as clouding agents, leading to widespread applications of these two phthalates in a variety of foods during the course of 15 years^7^. Since phthalates have been documented to have estrogenic effects as endocrine disruption compounds^8^, this raised concerns regarding the health effects of phthalate exposure on the people in Taiwan^9^.

Oxidative stress, a condition of increased reactive oxygen species, is now considered to be a feature of the normal aging process and many diseases^10^. Exposure to many environmental toxins, for example, bisphenol A^11^, polycyclic aromatic hydrocarbons^12^, and heavy metal^13^ have been linked to increase biomarkers of oxidative stress in human. In a number of cellular and animal studies, some phthalates, particularly DEHP, have been shown to cause increases in various markers of oxidative stress^14^. Multiple mechanisms, such as activation of peroxisome proliferator–activated receptors (PPAR) or increases in the permeability of mitochondrial membranes^15^,^16^, are potentially involved. In epidemiological studies using data from the National Health and Nutrition Examination Survey (NHANES), exposure to phthalates was associated with increased inflammation and indirect oxidative stress including serum levels of bilirubin, absolute neutrophil counts, alkaline phosphatase and ferritin levels^17^, C-reactive protein, gammaglutamyltransferase^18^. Another studies also found that exposure to phthalate is positively associated with biomarkers of oxidative stress, urinary malondialdehyde in the elderly population^19^, 8-hydroxydeoxyguanosine (8-OHdG) in women^20^, and 8-iso prostaglandin F2α (8-isoPGF2α) and 8-OHdG^21^,^22^ in pregnant women.

The total intake of phthalates, excluding non-dietary ingestion, is higher in all children than in adults^23^. As such, the exposure of the younger population to phthalates is greater than that of older adults. It remains unknown whether there is an association between phthalate exposure and various biomarkers of oxidative stress in adolescents and young adults. Given that a variety of methods have been proposed and used for the measurement of oxidative DNA, lipid, and protein damage in humans^10^, we used urine phthalate metabolites as the biomarkers for exposure to phthalates, urine 8-OHdG as biomarkers of DNA damage and 8-isoPGF2α as biomarkers of lipid peroxidation to perform a cross-sectional study of adolescents and young Taiwanese adults based on a nationwide mass urine screening.

## Materials and Methods

### Study population and data collection

**Figure 1:**
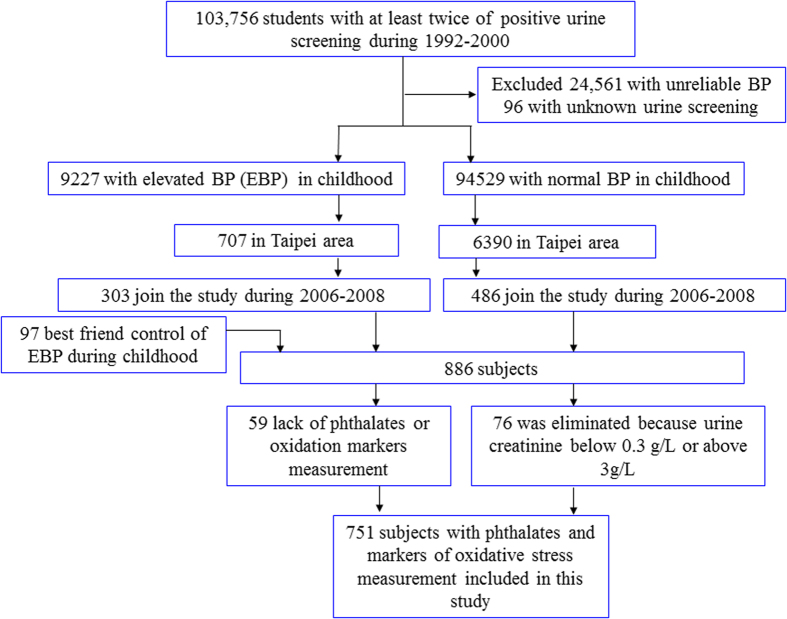
Algorithm used to select the participants.

The study population was composed of students who participated in the 1992–2000 mass urine-screening program in Taiwan^24^. Detailed information on the study subjects has been published before^25^. In the present study, a total 886 subjects were included in this study. The subjects were interviewed and given cardiovascular health check-ups at the National Taiwan University Hospital (NTUH) between 2006 and 2008. The study was approved by the Research Ethics Committee, NTUH. Written informed consent was obtained from each participant or from the parents of children and adolescents when they enrolled in the follow-up study. All methods in this study were performed in accordance with the relevant guidelines and regulations. A detailed flow chart of the selection process is shown in Fig. 1. Fifty-nine individuals were excluded because of unavailable urine samples for testing phthalates or oxidation of DNA and Lipid. Seventy-six subjects were eliminated as their urine creatinine levels were below 0.3 g/L or above 3 g/L, i.e., the World Health Organization recommended guidelines for acceptable variability of creatinine levels in urine specimens^26^. Ultimately, 751 participants were enrolled in the present study.

### Anthropometric and biochemical data

Socio-demographic information, such as age, gender, history of medication, and household income, was recorded during the interviews. Smoking status was subdivided into non-current smoker, <10 cigarettes/day, 10–19 cigarettes/day, and ≥20 cigarettes/day. Alcohol intake was determined by questionnaire and was categorized into two groups: “Current alcohol consumption” and “No alcohol consumption now”. Household income was categorized as either “above or below 50,000 new Taiwan dollars (NTD) per month.” Body mass index (BMI) was calculated as body weight divided by the square of body height (Kg/m^2^). Two seated blood pressure and heart rate measurements were made at least one minute apart after five minutes of rest, using a mercury manometer and appropriate cuff size. Hypertension status was determined by the self-reported current use of anti-hypertensive medication or an average BP ≥140/90 mmHg. Childhood elevated blood pressure was defined as either systolic blood pressure (SBP) or diastolic blood pressure, or both, that was greater than or equal to the modified sex- and age-specific criteria for blood pressure values^27^.

Subjects who fasted at least 8 hours or more were examined in the morning. The serum and urine samples were stored at −80 °C before analysis. The levels of urine creatinine, serum triglycerides (TG), low density lipoprotein cholesterol (LDL-C), and glucose were measured with an auto analyzer (Technician RA 2000 Autoanalyzer, Bayer Diagnostic, Mishawaka, IN). Serum insulin levels were measured with the commercial kit IMMULITE 2000 (Siemens Healthcare Diagnostics, Tarrytown, NY). The homeostasis model assessment of insulin resistance (HOMA-IR) index (the product of basal glucose and insulin levels divided by 22.5) is regarded as a simple, inexpensive, and reliable surrogate measure of insulin resistance^27^. Diabetes mellitus (DM) was defined as fasting serum glucose ≥126 mg/dL or self-reported current use of oral hypoglycemic agents or insulin.

### Measurements of Urine Metabolites of Phthalates

The urine samples were stored at −80 °C before analysis. Detailed information on the measurement has been published before^28^. Briefly, nine standard phthalate metabolites, including DEHP metabolites (mono(2-ethylhexyl) phthalate (MEHP), mono(ethyl-5-hydroxyhexyl) phthalate (MEHHP), mono(2-ethly-5-oxoheyl) phthalate (MEOHP)), di-methyl phthalate (DMP) metabolite (mono-methyl phthalate (MMP)), di-iso-butyl phthalate metabolite (mono-isobutyl phthalate (MiBP)), di-ethyl phthalate metabolite (mono-ethyl phthalate (MEP)), di-butyl phthalate metabolite (mono-n-butyl phthalate (MnBP)), butyl-benzyl phthalate metabolite (mono-benzyl phthalate (MBzP)), di-iso-nonyl phthalate metabolite (mono-iso-nonyl phthalate (MiNP)) and their corresponding isotopic ^13^C_4_-labeled compounds, were purchased from Cambridge Isotope Laboratories (Andover, MA, USA). The structures of the nine phthalates and their metabolites were available in the Supplementary Fig. S1. The chromatographic separation was performed using a Thermo Fisher-Accela UPLC system. The column was an Accucore C18 2.6 μm (2.1 × 100 mm) and was thermostatic at 25 °C. The mobile phases, consisting of 5% acetonitrile aqueous solution with 0.1% acetic acid (Mobile phase A) and 0.1% of acetic acid in acetonitrile (Mobile phase B), were delivered at a flow rate of 0.4 mL/min according to the following gradient: 0 min: (A)100%, 2 min: 80(A)/20(B)%, 17 min: 70(A)/30(B)%, 20 min: 20(A)/80(B)%, and 22 min: 80(A)/20(B)%. The column was re-equilibrated for 5 min. The MS/MS detection was performed using a ThermoFisher-TSQ Quantum Access Triple Quadruple LC–MS/MS with an ESI source operating in the negative ion mode. The spray voltage was −3500 V and the N_2_ sheath gas pressure was 45 psi. The N_2_ auxiliary gas pressure was 10 psi, the capillary temperature was 270 °C, and the collision gas (Ar) pressure was 1.5 mTorr. The multiple reactions monitoring condition was used to monitor the transition ion pairs, that is, m/z 179.0 → 107.4 and 179.0 → 77.0 for MMP, m/z 193.1 → 121.0 and 193.1 → 77.4 for MEP, m/z 221.0 → 121.0 and 221.0 → 77.4 for MiBP, m/z 221.1 → 121.3 and 221.1 → 77.4 for MnBP, m/z 293.1 → 145.3 and 293.1 → 121.0 for MEHHP, m/z 291.0 → 143.2 and 291.0 → 127.4 for MEOHP, m/z 255.1 → 183.0 and 255.1 → 107.3 for MBzP, m/z 277.2 → 134.2 and 277.2 → 127.2 for MEHP, and m/z 291.2 → 247.3 and 291.2 → 120.8 for MiNP, for qualification and quantification. Simultaneously, the mass transitions of the corresponding isotopic internal standards, i.e., m/z 183.0 → 79.0 for ^13^C_4_-MMP, m/z 197.1 → 79.0 for ^13^C_4_-MEP, m/z 225.1 → 79.0 for ^13^C_4_-MBP, m/z 297.1 → 124.0 for ^13^C_4_-MEHHP, m/z 295.1 → 124.0 for ^13^C_4_-MEOHP, m/z 259.1 → 107.0 for ^13^C_4_-MBzP, m/z 281.2 → 137.0 for ^13^C_4_-MEHP, and m/z 295.2 → 141.0 for ^13^C_4_-MiNP, were also monitored. The total analysis time was 22 min for each sample. The limits of detection (LOD), defined as the analyte concentration required to produce a signal greater than three times the standard deviation of the noise level, for three urinary phthalate metabolites were 1.0 ng/mL (MiBP, MnBP, MiNP) and 0.5 ng/mL for the others. The pronounced R squared of the calibration curve was higher than 0.995. Method accuracy and precision was evaluated using QC samples at low (5 ng/mL), medium (25 ng/mL), and high (50 ng/mL) concentrations. The accuracy calculated as the percentage error ranged from 92.20–124.23%. The precision expressed as the coefficient of variation was less than 0.5%.

### Measurement of urinary levels of 8-OHdG

8-OHdG was measured by LC-MS/MS, as described elsewhere^29^,^30^. Briefly, urine was collected in glass bottles and processed immediately or stored at −20 °C. For processing, 20 μL of urine was diluted 20-fold with 5% MeOH (v/v) and 0.1% formic acid, spiked with 50 μL of ^15^N_5_-8-OHdG (1 ng/mL), and mixed by vortexing. The samples were analyzed using a column-switching system with a switching valve (10-port, 2-position microelectric actuator from Valco Instrument Co., Ltd.) and an Inertsil ODS-3 (33 × 4.6 mm × 5 μm) column. The switching valve function was controlled by Analyst 1.4.2 software (AB SCIEX, Canada). The HPLC system consisted of a binary pump, an autosampler (Agilent 1260VL, Agilent Technology, USA), and a ZORBAX Eclipse Plus C18 3.5 μm, 100 mm × 4.6 mm column. The eluted samples were introduced into a TurboIonSpray source using an electrospray ionization probe installed on an API 3000 triple-quadruple mass spectrometer (AB SCIEX) operated in the positive mode with a needle voltage of 4500 V. The optimized source parameter multiple reaction monitoring mode transition pairs of 8-OHdG and ^15^N_5_-8-OHdG were set as m/z 284 → 168 m/z and 289 → 173 for the quantitative pair and m/z 284 → 140 and m/z 289 → 145 for the qualitative pair, respectively. Data acquisition and quantitative processing were accomplished using Analyst 1.4.2 software. Satisfactory recovery was obtained; the recovery was 96.7% in the urine, with a coefficient of variation less than 1.9% (n = 5). The values for the intra-day and inter-day precision were between 2.7 and 4.5% for urine; the values for the method accuracy of the intra-day and inter-day assays ranged from 110.2 and 119.4% for urine (n = 5). The LOD for 8-OHdG was 0.02 ng/mL. All laboratory analyses were conducted by investigators blinded to the characteristics of the study subjects.

### Measurement of urinary levels of 8-isoPGF2α

The four stock standard solutions were prepared to 100 mg/L for each, and they were stored in polypropylene screw-top tubes at −20 °C. To evaluate the amounts of analytes in the urine, a set of 8-isoPGF_2α_ standard solutions was diluted from 0.5 to 50.0 ng/mL with 50% ACN + 0.1% FA (v/v) and was spiked with a fixed amount of 50 ng/mL 8-isoPGF-d_4_ as internal standards. An 800 μL aliquot of urinary sample was spiked 50.0 ng mL^−1^ internal standards prior to vortex mixing. During the solid phase extraction, the residue was reconstituted in 50 μL of 50% ACN + 0.1% FA (v/v) and then the sample was frozen at −20 °C until it could be analyzed. The online extraction system consisted of an extraction column (Inertsil ODS-3 33 mm × 4.6 mm, 5 μm) and a 10-port switching valve (2-position micro electric actuator from Valco Instrument Co., Ltd.). The switching valve function was controlled by Analyst 1.4.2^TM^ software (AB SCIEX, Canada). Analytical separation of these lipidomic metabolites used a Waters dC18 column (Waters dC18, 100 mm × 2.1 mm, 5 μm) with a binary gradient system composed of mobile phase A (1% ACN, v/v with 0.1% FA) and mobile phase B (90% ACN, v/v with 0.1% FA) at a flow rate of 0.5 mL min^−1^. The gradient was started at 85% mobile phase A, which was maintained for 3.8 min, ramped to the 30% mobile phase B in 0.7 min, then ramped to 10% mobile phase B in 20 min, and held for 2.7 min at 85% mobile phase A to finish the 25 min cycle of analysis before the next sample was injected. The mass spectrometric data were collected using a triple-quadrupole mass spectrometer, API 3000 (Applied Biosystems, MDS SCIEX, Concord, Ontario, Canada), operating with a TurboIonSpray source. The mass spectrometer coupled with electrospray ionization (ESI) interfaces was operated in negative ion mode at an ion spray voltage of −4000 V. Optimization results for each analyte in multiple reaction monitoring scan mode. The source settings were as follows: nebulizer gas at 13 psi, curtain gas at 10 psi, collision-assisted dissociation at 8 psi, and the source heater probe temperature at 500 °C. The optimized source parameter multiple reaction monitoring mode transition pairs of 8-isoPGF2α and 8-isoPGF2α-d4 were set as m/z 353 → 193 and m/z 357 → 197.

Using on-line solid-phase extraction for the determination of the four metabolites was effective and rapid. The precision and accuracy were calculated by spiking into urine at three different known levels (2.5, 10.0, and 25.0 ng/mL), and the accuracy was expressed as accuracy (%) = [(measured concentration − blank urine matrix concentration)/spiked concentration] × 100%. The accuracy (n = 3) of the ten metabolites ranged from 81.49–120.12%. The precision of the procedure was demonstrated by spiking urine matrix samples with three concentrations (2.5, 10.0, and 25.0 ng/mL) of standards to evaluate inter-day and intra-day assay precision. These preparations were also used as quality control samples to monitor the day-to-day performance of the assays. The intra-day (n = 3) and inter-day (n = 9) assay variations were 0.64–18.07% and 3.04–20.91%, respectively. The matrix effects in the present study were calculated by comparing the peak areas of a known concentration of internal standards in a neat solvent and in the spiked urine samples after extraction. The recovery was expressed as recovery (%) = [(A_IS after extraction from urine_)/(A_IS in neat solvent_)] × 100%. The results show that the recovery ranged from 25.03–96.71%. The LOD for 8-isoPGF2α was 0.003 ng/mL.

### Statistical analysis

SPSS for Windows (version 20.0, SPSS Inc., Chicago, IL) was used for all of the statistical analyses. Because over 80% of the MiNP were below the LOD, we analyzed levels of other eight phthalate metabolites in this study. The urine phthalate metabolites, 8-OHdG, and markers of lipid peroxidation concentrations were expressed as geometric means with 95% confidence intervals. For concentrations below the LOD, a value of half the LOD was used. The three DEHP metabolites, i.e., MEHP, MEHHP and MEOHP, were combined into one measure in molar concentrations for analysis, denoted by ΣMEHP due to their high correlation and similar characteristics. Levels of ∑MEHP were the sum of (MEHP/278) + (MEHHP/294) + (MEOHP/292) and corrected for urine creatinine^31^. Other phthalate metabolites, 8-OHdG, and 8-isoPGF2α were corrected for urine creatinine and expressed as μg/g creatinine. The relationship between the phthalate metabolites and the categorical variables was tested using the Mann-Whitney U test or the Kruskal-Wallis Test (if there were three or more groups).

We used an extended model approach for the covariates to adjust for potential confounders in the multiple linear regression models to study the association between phthalate metabolites, 8-OHdG, and markers of lipid peroxidation. Model 1 adjusted for age and gender. Model 2 adjusted for age, gender, and other risk factors (smoking status, BMI, SBP, LDL-C, TG, and HOMA-IR). To avoid a “model-dependent association,” an association was only considered significant when it was statistically significant in both models^32^,^33^. Each metabolite was modelled separately in separate analyses. A natural log transformation, performed for urinary phthalate metabolite levels, 8-OHdG, 8-isoPGF2α, TG and HOMA-IR, showed significant deviation from the normal distribution before further analysis. We used the Bonferroni correction to correct the multiple comparisons and testing. Because we tested six types of phthalate metabolites at the same time in our study, a *P* value < 0.0083 (0.05 divided by six) was considered significant when we investigated the association between phthalate metabolites, 8-OHdG, and markers of lipid peroxidation.

## Results

The study sample consisted of 302males and 449 females. The mean age was 21.45 years old. The median (25^th^ and 75^th^ percentile) concentrations of urine phthalate metabolites were MEHP: 11.35(1.29–34.97) μg/g creatinine, MEHHP: 23.84(15.33–40.68) μg/g creatinine, and MEOHP: 15.16(9.73–25.25) μg/g creatinine; ∑MEHP: 0.20(0.12–0.38) μmol/g creatinine; MMP: 7.56(4.84–11.97) μg/g creatinine; MEP: 29.44(12.47–72.09) μg/g creatinine; MiBP: 15.13 (8.98–26.46) μg/g creatinine; MnBP: 36.22 (21.79–61.01) μg/g creatinine; and MBzP: 1.77(0.95–3.40) μg/g creatinine. The geometric means and 95% confidence intervals of the concentrations of the creatinine adjusted urinary phthalate metabolites in different subpopulations are shown in Table 1. After comparing by the Mann-Whitney U test, the younger age group (12–19-year-olds) had a significantly higher concentration of MiBP than the older age group, while females had a significantly higher concentration of ∑MEHP, MMP, MiBP, MEP, and MnBP than males. Moreover, subjects with lower serum triglyceride (<160 mg/dL) had a significantly higher concentration of MMP and MEP than the subjects with higher triglyceride. The phthalate metabolite concentrations were not different between the other subpopulations. The geometric means and 95% confidence intervals of the concentrations of the creatinine adjusted urinary 8-OHdG and 8-isoPGF2α in different subpopulations are shown in Table 2. The older age group (20–30-year-olds) had a significantly higher concentration of 8-isoPGF2α than the younger age group. There were positive association between smoking amount and concentration of 8-OHdG. Subjects with DM had a significantly higher 8-isoPGF2α than those without DM. The 8-OHdG concentration was not different between the other subpopulations. The geometric means and 95% confidence intervals of the concentrations (not adjusted by urine creatinine) of the urinary phthalate metabolites, 8-OHdG and 8-isoPGF2α in different subpopulations are shown in Supplementary Tables S2 and S3.

Linear regression coefficients of 8-OHdG and 8-isoPGF2α with a unit increase in urine creatinine adjusted phthalate metabolites are shown in Table 3. The concentrations of ∑MEHP, MiBP, MnBP and MBzP were not associated with the urine levels of the 8-OHdG and 8-isoPGF2α. However, MMP was positively associated with urine levels of the 8-OHdG and 8-isoPGF2α.

Linear regression coefficients of markers of oxidative stress with a unit increase in urine creatinine-adjusted MMP in subpopulations of the sample subjects are shown in Table 4. The association between MMP and 8-OHdG was significant in individuals older than 20 years old, with male gender, lower BMI, higher HOMA-IR, higher systolic blood pressure and triglyceride, and patient without DM. The strength of the association between the level of MMP and 8-isoPGF2α appeared to be statistically significant in almost all subpopulations (Except subjects with DM).

## Discussion

This cross-sectional study in adolescents and young adults demonstrates a positive association between urinary MMP levels and markers of oxidative stress to DNA and lipids. Exposures to DMP, but not other phthalates, were associated with increased oxidative stress. This is the first clinical report to link urine phthalate metabolites and biomarkers of oxidative damage to DNA and lipids in younger population.

Except for MBzP, results showed that female subjects had significantly higher phthalate metabolite concentrations in urine comparing by the Mann-Whitney U test. Females may have a greater chance of exposure to phthalate because of the use of cosmetics and other consumer products in which phthalate has been detected^34^. We reported a median (25^th^ and 75^th^ percentile) MEHP concentration of 20.87 (6.50–56.50) μg/g creatinine in participants aged between 12 to 15 in this study, which is similar with another cohort which MEHP concentration were 29.8 (13.1–72.8) μg/g creatinine in children aged between 12 to 15 in Taiwan^9^. This level is higher than other literatures for MEHP levels in the United States^35^ and Canada^36^, at 3.00 and 2.70 μg/g creatinine, respectively. In May 2011, the Taiwan Food and Drug Administration reported that the plasticizer DEHP and di-iso-nonyl phthalate were illegally added to clouding agents used in foods and beverages^7^,^9^. Higher DEHP exposure in our study population may have been a r‘12esult of this scandal because the samples were collected from 2006 to 2008^28^. However, it is recognized that MiNP, secondary metabolites of di-iso-nonyl phthalate, is not found often in urine samples in this study. This finding is compatible with the report from NHANES in the US of which shows occurrence frequency of this metabolite at less than 10%^37^. Because of this, practitioners have recently taken to measure a secondary metabolite, mono-(carboxyoctyl) phthalate as an alternative^38^.

Considerable attention has focused on identifying suitable biomarkers to assess *in vivo* rates of oxidative damage. Candidate biomarkers can be classified into three major groups: markers of oxidative damage to lipids, proteins, and nucleic acids. We selected 8-OHdG and 8-isoPGF2α for measurement in this study not only because of their well-documented usefulness as systemic biomarkers of oxidative stress for establishing associations with adverse health outcomes^39^ but also because of their representation of different cellular reactions to ROS exposure and the potential downstream effects of the biomarkers themselves. 8-isoPGF2α, generated non-enzymatically by free radical-mediated oxidation of arachidonic acid, is probably the most popular and recommended eicosanoid molecule used as a biomarker for oxidative stress. Urinary 8-OHdG levels may be confounded by differences in the DNA repair capacity^39^.

An unexpected finding in the current study was that MMP, a urine metabolite of DMP, was strongly correlated with biomarkers of oxidative stress. In rodent studies, activation of PPARαwas induced by several phthalates and its metabolites, especially MEHP. PPARγ, which leads to adipocyte differentiation and insulin sensitization, was also activated by MEHP. However, this phenomenon was not observed with the DMP or MMP^40^. A biological mechanism that might result in positive effects of phthalates on oxidative stress has not been established. In aquatic environment, most water organic pollutants may form more toxic byproducts than their parent compounds during the photochemical transformation. The hydroxyl radical (•OH) is one of the main oxidative species in aqueous phase advanced oxidation processes^41^. A recent research investigated eco-toxicity and human estrogenic exposure risks of four phthalates (DMP, diethyl phthalate, dipropyl phthalate and dibutyl phthalate) and transformation products during the OH-initiated photochemical process. Kinetics analysis revealed that OH-addition transformation products are mainly formed for DMP, which have greater aquatic toxicity than other three phthalates^42^. In humans, unlike DEHP which has been widely studied, DMP and its urine metabolite MMP were not included as biomarkers of phthalate exposure when investigating the association between phthalate exposure and biomarkers of oxidative stress in most previous epidemiological studies^17^,^18^,^19^,^21^,^22^. In a longitudinal study designed to assess the relationship between environmental chemicals exposure and fecundity in couples who were planning pregnancy, significant relationships were found between 8-OHdG and MEP, MEHP, and MiBP, while insignificant associations were found between 8-OHdG and MMP^20^. This discrepancy in results among various epidemiological studies could be attributable to differences in diet, product use, toxicant metabolism, and/or other differences between study populations^22^. However, DMP is one of the most frequently used phthalates. The impact of this chemical on the environment and its toxicity to living organisms is of great concern^43^. In animals and humans, increasing evidence suggests that lower molecular weight phthalates, such as DMP and its metabolites, may interfere with the development and reproductive systems^44^,^45^. Moreover, DMP and its intermediates have been reported to exhibit teratogenicity, mutagenicity and cellular toxicity^46^. Findings in the current study strongly suggest that more toxicological studies on DMP need to be performed.

There are several limitations in the current study. First, the patient population of our study consists of adolescents and young adults with an abnormal urinalysis in childhood and living in the Taipei area; therefore, we cannot infer that the same result might be observed in the general population. Second, we did not consider all of the medications that might have had an impact on phthalate metabolites and markers of oxidative stress, which would be a confounding variable. However, more than 95% of the participants self-reported no significant clinical diseases and no medication history. Third, there was potential bias for taking first morning samples instead of samples taken randomly throughout the day. For phthalates like DEHP, butyl-benzyl phthalate, the highest concentrations of its metabolites have been found in the early evening; while for phthalates metabolites like MEP, the elevated levels have been observed in the midday collection samples^47^,^48^. Forth, we did not include other environmental factors (for example, bisphenol A, a potentially harmful chemical in plastic containers) that may be important confounders or explanatory variables for the outcomes of our study^25^. Finally, since this study was a cross-sectional study, it is not possible to infer causality.

We found a significant positive association between urine DMP metabolite, MMP, and biomarkers of oxidative stress in Taiwanese adolescents and young adults. This association appears to be significant after controlling traditional risk factors as covariates such as age, gender, smoking, BMI, HOMA-IR, blood pressure, and serum lipid levels by multiple linear regression model. If a causal relationship exists, DMP exposure may cause acute or chronic diseases by inducing oxidative damage to DNA and lipids. Future epidemiological and toxicological research on exposure to DMP and oxidative stress is warranted.

## Additional Information

**How to cite this article:** Lin, C.-Y. *et al*. Positive Association between Urinary Concentration of Phthalate Metabolites and Oxidation of DNA and Lipid in Adolescents and Young Adults. *Sci. Rep.* **7**, 44318; doi: 10.1038/srep44318 (2017).

**Publisher's note:** Springer Nature remains neutral with regard to jurisdictional claims in published maps and institutional affiliations.

## Supplementary Material

Supplementary Information

## Figures and Tables

**Table 1:** Basic demographics of the sample subjects including geometric means and their 95% confidence intervals of the creatinine adjusted urinary phthalate metabolites.

	No.	∑MEHP (μmol/g creatinine)	MMP (μg/g creatinine)	MiBP (μg/g creatinine)	MEP (μg/g creatinine)	MnBP (μg/g creatinine)	MBzP (μg/g creatinine)
Overall	751	0.22 (0.21–0.24)	7.51 (7.13–7.90)	15.09 (14.04–16.22)	31.21 (28.58–34.09)	35.52 (33.27–37.23)	1.93 (1.78–2.09)
Age							
12–19	217	0.24 (0.21–0.27)	7.72 (7.01–8.50)	17.23* (15.07–19.69)	27.86 (23.67–32.82)	37.38 (33.08–42.22)	1.83 (1.58–2.12)
20–30	534	0.22 (0.20–0.24)	7.42 (6.98–7.89)	14.30* (13.13–15.56)	32.68 (29.43–36.27)	34.79 (32.20–37.60)	1.97 (1.79–2.16)
Gender							
Female	449	0.25** (0.23–0.27)	8.07** (7.55–8.63)	16.77** (15.29–18.39)	41.47** (37.19–46.25)	39.30** (36.13–42.73)	2.02 (1.82–2.23)
Male	302	0.19** (0.17–0.21)	6.73** (6.21–7.30)	12.89** (11.52–14.49)	20.44** (17.89–23.36)	30.55** (27.58–33.85)	1.80 (1.59–2.04)
Household income							
<50000 NTD per month	292	0.23 (0.20–0.26)	7.31 (6.73–7.94	14.55 (12.96–16.35)	29.63 (25.74–34.12)	34.71 (31.25–38.55)	1.82 (1.60–2.07)
≥50000 NTD per month	458	0.22 (0.20–0.24)	7.64 (7.15–8.16)	15.44 (14.07–16.93)	32.39 (28.93–36.27)	36.09 (33.18–39.25)	2.00 (1.81–2.21)
Smoking status							
Non-current smoker	622	0.23 (0.21–0.25)	7.57 (7.16–8.00)	15.35 (14.20–16.59)	32.59 (29.64–35.84)	36.23 (33.75–38.90)	1.95 (1.79–2.13)
<10 cigarettes/day	47	0.22 (0.17–0.29)	7.49 (6.09–9.21)	14.30 (10.72–19.07)	30.63 (21.54–43.55)	30.05 (23.13–39.02)	1.86 (1.35–2.56)
10–19 cigarettes/day	48	0.20 (0.15–0.26)	7.19 (5.86–8.82)	12.38 (9.31–16.46)	23.47 (16.58–33.28)	32.02 (24.85–41.72)	1.76 (1.28–2.41)
≥20 cigarettes/day	34	0.18 (0.13–0.25)	7.84 (6.15–9.99)	14.28 (10.19–20.05)	22.94 (15.17–34.71)	27.55 (20.23–37.49)	1.89 (1.30–2.76)
Current alcohol consumption							
No	682	0.22 (0.21–0.24)	7.42 (7.03–7.83)	14.83 (13.75–15.99)	31.09 (28.33–34.12)	35.20 (32.85–37.71)	1.91 (1.75–2.07)
Yes	69	0.21 (0.17–0.27)	8.46 (7.13–10.02)	17.90 (14.11–22.69)	32.38 (24.19–43.29)	38.85 (31.28–48.23)	2.12 (1.63–2.75)
Body mass index (kg/m^2^)							
<24	588	0.22 (0.20–0.23)	7.46 (7.04–7.90)	14.83 (13.67–16.09)	32.50 (29.43–35.91)	33.65 (29.43–35.91)	1.83 (1.67–2.00)
≥24	163	0.25 (0.22–0.29)	7.69 (6.88–8.59)	16.06 (13.76–18.74)	26.95 (22.31–32.56)	43.18 (22.31–32.56)	2.34 (1.97–2.77)
Hypertension							
No	690	0.22 (0.21–0.24)	7.62 (7.22–8.04)	14.95 (13.86–16.12)	31.94 (29.14–35.02)	35.29 (32.95–37.79)	1.94 (1.78–2.10)
Yes	61	0.24 (0.19–0.31)	6.32 (5.28–7.57)	16.80 (13.05–21.63)	23.97 (17.60–32.66)	38.24 (30.39–48.13)	1.82 (1.37–2.40)
DM							
No	735	0.22 (0.20–0.24)	7.49 (7.11–7.89)	14.95 (13.90–16.07)	31.11 (28.45–34.02)	35.21 (32.95–37.64)	1.92 (1.77–2.08)
Yes	16	0.36 (0.22–0.59)	8.16 (5.73–11.62)	23.33 (14.18–38.02)	35.84 (19.59–65.56)	52.92 (33.78–82.85)	2.30 (1.33–3.96)
LDL-C(mg/dL)							
<130	626	0.22 (0.21–0.24)	7.55 (7.13–7.98)	15.00 (13.86–16.23)	31.94 (29.02–35.20)	35.16 (32.72–37.79)	1.94 (1.78–2.11)
≥130	125	0.23 (0.20–0.27)	7.32 (6.46–8.31)	15.55 (13.03–18.54)	27.74 (22.35–34.43)	37.34 (31.79–43.86)	1.87 (1.54–2.27)
Triglyceride(mg/dL)							
<160	715	0.22 (0.21–0.24)	7.60* (7.21–8.02)	15.04 (13.97–16.20)	31.84* (29.11–34.85)	35.23 (32.92–37.68)	1.89 (1.74–2.05)
≥160	36	0.26 (0.19–0.37)	5.75* (4.55–7.28)	16.02 (11.53–22.26)	20.78* (13.92–31.06)	42.01 (31.12–56.66)	2.80 (1.95–4.03)

^*^P < 0.05.

**P < 0.01.

^∑^MEHP, sum of (MEHP/278) + (MEHHP/294) + (MEOHP/292).

**Table 2:** Basic demographics of the sample subjects including geometric means and their 95% confidence intervals of the creatinine adjusted urinary 8-OHdG and 8-isoPGF2α.

	No.	8-OHdG (μg/g creatinine)	8-isoPGF_2α_ (μg/g creatinine)
Overall	751	2.02 (1.94–2.10)	0.87 (0.81–0.94)
Age			
12–19	217	1.97 (1.84–2.12)	0.68** (0.60–0.78)
20–30	534	2.03 (1.94–2.13)	0.97** (0.89–1.05)
Gender			
Female	449	1.99 (1.89–2.09)	0.91 (0.83–1.00)
Male	302	2.06 (1.93–2.19)	0.82 (0.73–0.92)
Household income			
<50000 NT dollars per month	292	2.02 (1.90–2.15)	0.84 (0.75–0.94)
≥50000 NT dollars per month	458	2.01 (1.91–2.11)	0.90 (0.82–0.99)
Smoking status			
Non-current smoker	622	1.99* (1.91–2.08)	0.85 (0.79–0.92)
<10 cigarettes/day	47	1.75* (1.50–2.04)	0.96 (0.72–1.28)
10–19 cigarettes/day	48	2.21* (1.89–2.58)	0.94 (0.71–1.25)
≥20 cigarettes/day	34	2.47*(2.05–2.96)	1.12 (0.80–1.57)
Current alcohol consumption			
No	682	1.99 (1.91–2.08)	0.88 (0.81–0.95)
Yes	69	2.23 (1.96–2.54)	0.84 (0.66–1.06)
Body mass index (kg/m^2^)			
<24	588	2.04 (1.95–2.13)	0.85 (0.79–0.92)
≥24	163	1.93 (1.77–2.09)	0.96 (0.82–1.12)
Hypertension			
No	690	2.01 (1.93–2.10)	0.87 (0.80–0.93)
Yes	61	2.03 (1.77–2.33)	0.98 (0.76–1.26)
DM			
No	735	2.01 (1.94–2.09)	0.86** (0.80–0.93)
Yes	16	2.12 (1.62–2.77)	1.69** (1.03–2.77)
LDL-C(mg/dL)			
<130	626	2.03 (1.94–2.11)	0.86(0.80–0.94)
≥130	125	1.96 (1.78–2.15)	0.93(0.78–1.12)
Triglyceride(mg/dL)			
<160	715	2.02(1.94–2.10)	0.87(0.81–0.94)
≥160	36	1.89(1.58–2.26)	0.98(0.71–1.37)

*P < 0.05.

**P < 0.01.

**Table 3:** Linear regression coefficients (standard error) of 8-OHdG and 8-isoPGF_2α_ with a unit increase in natural log-transformed phthalate metabolites in multiple linear regression models (n = 751).

	Ln 8-OHdG (μg/g creatinine)	P value	Ln 8-isoPGF_2α_ (μg/g creatinine)	P value
Ln ∑MEHP (μmol/g creatinine)				
Model 1	0.000(0.020)	0.997	0.070(0.037)	0.060
Model 2	0.008(0.020)	0.694	0.065(0.037)	0.081
Ln MMP (μg/g creatinine)				
Model 1	0.098(0.028)	<0.001	0.251(0.050)	<0.001
Model 2	0.096(0.028)	0.001	0.253(0.051)	<0.001
Ln MiBP (μg/g creatinine)				
Model 1	0.044(0.020)	0.029	0.069(0.037)	0.063
Model 2	0.044(0.020)	0.026	0.064(0.037)	0.084
Ln MEP (μg/g creatinine)				
Model 1	0.024(0.017)	0.152	−0.005(0.031)	0.869
Model 2	0.021(0.017)	0.215	−0.003(0.031)	0.914
Ln MnBP (μg/g creatinine)				
Model 1	0.028(0.022)	0.202	0.006(0.040)	0.878
Model 2	0.033(0.022)	0.141	−0.004(0.041)	0.914
Ln MBzP (μg/g creatinine)				
Model 1	0.024(0.018)	0.178	0.076(0.033)	0.022
Model 2	0.022(0.018)	0.227	0.073(0.033)	0.029

∑MEHP, sum of (MEHP/278) + (MEHHP/294) + (MEOHP/292).

Model 1: adjusted for age and gender.

Model 2: adjusted for age, gender and other risk factors (smoking status, body mass index, systolic blood pressure, low density lipoprotein, triglyceride, and homeostasis model assessment of insulin resistance).

**Table 4:** Linear regression coefficients (standard error) of natural log transformed markers of oxidative stress with a unit increase in natural log transformed MMP (creatinine adjusted) in subpopulations of the sample subjects.

	N	Ln 8OHdG(μg/g creatinine)		Ln 8-isoPGF_2α_ (μg/g creatinine)	
		Coefficients (S.E.)	*P* value	Coefficients (S.E.)	*P* value
Age, yr					
<20	217	0.086(0.043)	0.050	0.353(0.090)	<0.001
≥20	534	0.091(0.035)	0.009	0.222(0.061)	<0.001
Sex					
Female	449	0.040(0.034)	0.237	0.121(0.070)	0.003
Male	302	0.174(0.046)	<0.001	0.320(0.073)	<0.001
BMI, kg/m^2^					
<20.95	376	0.141(0.037)	<0.001	0.198(0.074)	0.007
≥20.95	375	0.052(0.041)	0.200	0.306(0.070)	<0.001
HOMA-IR					
<0.90	375	0.055(0.039)	0.156	0.387(0.077)	<0.001
≥0.90	376	0.117(0.039)	0.003	0.167(0.068)	0.015
Smoking status					
Not active smoker	644	0.083(0.030)	0.006	0.214(0.056)	<0.001
Active smoker	107	0.189(0.077)	0.016	0.412(0.121)	0.001
Systolic blood pressure					
<107	371	0.031(0.034)	0.362	0.257(0.074)	<0.001
≥107	380	0.160(0.043)	<0.001	0.251(0.069)	<0.001
LDL-C					
<99	374	0.068(0.031)	0.032	0.267(0.068)	<0.001
≥99	377	0.122(0.046)	0.008	0.231(0.077)	0.003
Triglyceride					
<70	375	0.073(0.044)	0.099	0.228(0.076)	0.003
≥70	376	0.101(0.035)	0.004	0.276(0.070)	<0.001
DM					
No	735	0.089(0.028)	0.001	0.2526(0.051)	<0.001
Yes	16	0.112(0.449)	0.539	0.078(0.979)	0.802

Model adjusted for age, gender and other risk factors (smoking status, body mass index, systolic blood pressure, low density lipoprotein, triglyceride, and homeostasis model assessment of insulin resistance.
