# Supplementary material for: Positive Association between Urinary Concentration of Phthalate Metabolites and Oxidation of DNA and Lipid in Adolescents and Young Adults
Source: Sci Rep. 2017 Mar 14;7:44318. doi: 10.1038/srep44318 (PMC5349565; doi:10.1038/srep44318)
Supplement: Supplementary Information [file srep44318-s1.pdf]

Supplementary Information

Title: Positive Association between Urinary Concentration of Phthalate Metabolites and Oxidation of DNA and Lipid in Adolescents and Young Adults

Chien-Yu Lin, Pau-Chung Chen, Chia-Jung Hsieh, Chao-Yu Chen, Anren Hu, Fung-Chang Sung, Hui-Ling Lee, and Ta-Chen Su

Supplementary Figure S1: The structures of the nine phthalates and their metabolites

No.	Phthalates	Metabolites
1	Dimethyl phthalate (DMP) 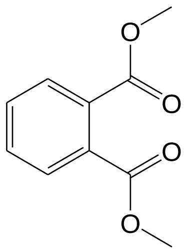	Mono-methyl phthalate (MMP) 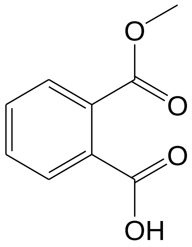
2	Diethyl phthalate (DEP) 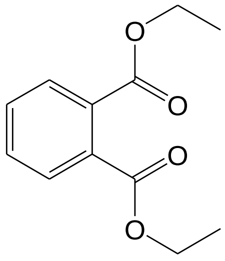	Monoethyl phthalate (MEP) 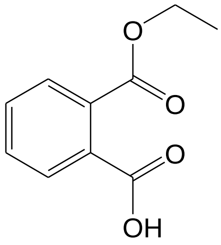
3	Dibutyl phthalate (DBP) 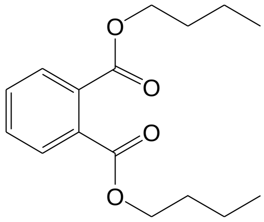 Butylbenzyl phthalate (BBzP) minor	Mono-n-butyl phthalate (MnBP) 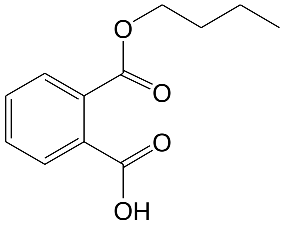

	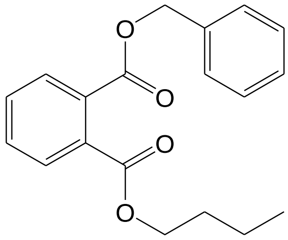	
4	Di-isobutyl phthalate (DiBP)<br>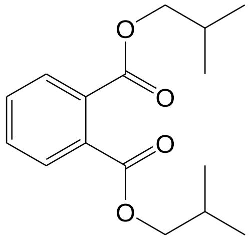	Mono-isobutyl phthalate (MiBP)<br>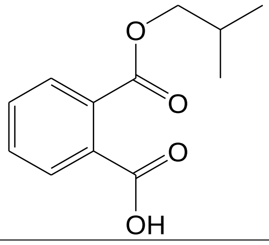
5	Di-2-ethylhexyl phthalate (DEHP)<br>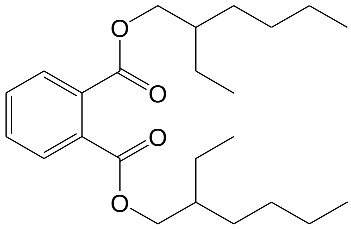	Mono-2-ethylhexyl phthalate (MEHP)<br>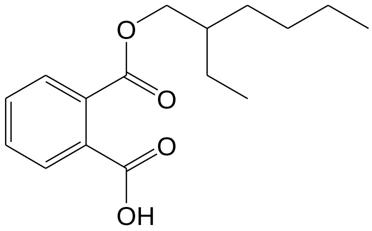
6	Di-2-ethylhexyl phthalate (DEHP)<br>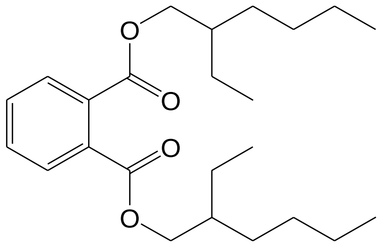	Mono-2-ethyl-5-hydroxyhexyl phthalate (MEHHP)<br>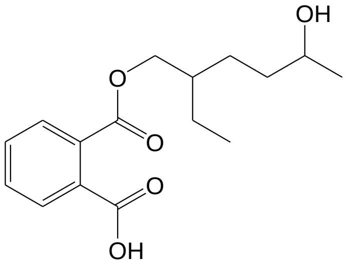
7	Di-2-ethylhexyl phthalate (DEHP)<br>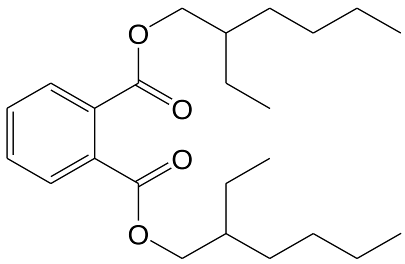	Mono-2-ethyl-5-oxo-hexyl phthalate (MEOHP)<br>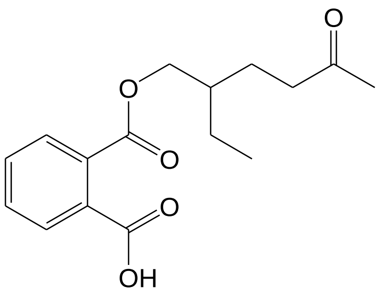
8	Diisononyl phthalate (DINP)	Monoisononyl phthalate (MiNP)

	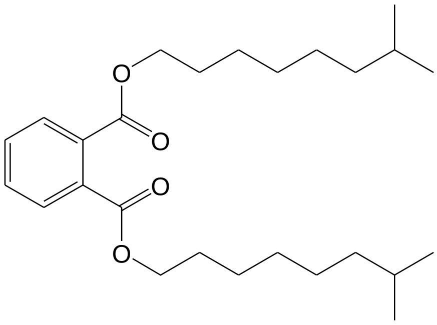	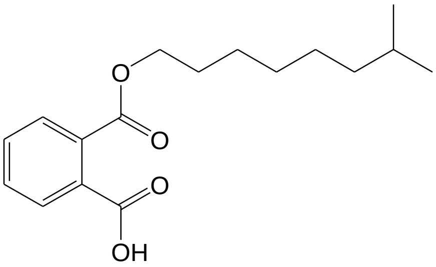
9	Butylbenzyl phthalate (BBzP)<br>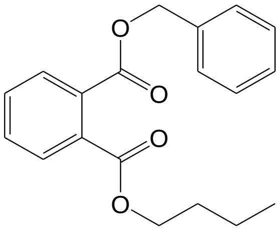	Monobenzyl phthalate (MBzP)<br>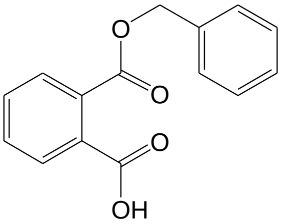

Supplementary Table S2:. Basic demographics of the sample subjects including geometric means and their 95% confidence intervals of the urinary phthalate metabolites

	No.	∑MEHP (µmol/L)	MMP (µg/L)	MiBP (µg/L)	MEP (µg/L)	MnBP (µg/L)	MBzP (µg/L)
Overall	751	0.33	10.98	22.07	45.64	51.95	2.82
		(0.30-0.35)	(10.37-11.62)	(20.35-23.97)	(41.69-49.74)	(47.97-55.67)	(2.58-3.08)
Age							
12-19	217	0.37*	11.87	26.50**	42.84	57.47	2.81
		(0.32-0.42)	(10.67-13.21)	(22.92-30.63)	(35.98-51.01)	(50.20-65.83)	(2.39-3.30)
20-30	534	0.31*	10.63	20.49**	46.83	49.86	2.82
		(0.28-0.34)	(9.93-11.38)	(18.67-22.47)	(41.89-52.35)	(45.74-54.33)	(2.54-3.12)
Gender							
Female	449	0.33	10.59	21.99	54.38**	51.54	2.64
		(0.30-0.36)	(9.83-11.40)	(19.87-24.34)	(48.23-61.31)	(46.90-56.66)	(2.36-2.96)
Male	302	0.32	11.58	22.17	35.17**	52.56	3.10
		(0.29-0.36)	(10.58-12.68)	(19.59-25.08)	(30.39-40.69)	(46.85-58.97)	(2.70-3.55)
Household income							
<50000 NTD per month	292	0.34	10.89	21.70	44.19	51.75	2.72
		(0.30-0.38)	(9.93-11.95)	(19.14-24.61)	(38.02-51.37)	(46.06-58.15)	(2.37-3.12)
≥50000 NTD per month	458	0.32	11.02	22.28	46.76	52.10	2.89
		(0.29-0.35)	(10.24-11.87)	(20.15-24.63)	(41.47-52.72)	(47.47-57.23)	(2.58-3.23)
Smoking status							
Non-current smoker	622	0.33	10.92	22.43	47.23	53.43	2.83

		(0.31-0.36)	(10.25-11.63)	(20.57-24.46)	(42.61-52.35)	(49.30-57.86)	(2.57-3.11)
< 10 cigarettes/day	47	0.35	11.98	22.86	48.96	48.03	2.98
		(0.26-0.47)	(9.51-15.09)	(16.71-31.28)	(33.68-71.16)	(35.91-64.20)	(2.10-4.21)
10–19 cigarettes/day	48	0.31	11.41	19.66	37.29	51.14	2.79
		(0.24-0.42)	(9.08-14.34)	(14.41-26.79)	(25.76-54.00)	(38.36-68.17)	(1.98-3.93)
≥ 20 cigarettes/day	34	0.23	10.06	18.34	29.45	35.38	2.43
		(0.16-0.32)	(7.67-13.20)	(12.69-26.50)	(18.97-45.70)	(25.13-49.80)	(1.62-3.65)
Current alcohol consumption							
No	682	0.33	10.83	21.66	45.41	51.41	2.79
		(0.30-0.35)	(10.20-11.51)	(19.95-23.50)	(41.14-50.10)	(47.61-55.48)	(2.54-3.05)
Yes	69	0.32	12.54	26.54	48.01	57.61	3.14
		(0.25-0.40)	(10.37-15.17)	(20.51-34.36)	(35.23-65.43)	(45.29-73.26)	(2.36-4.18)
Body mass index (kg/m^2)							
<24	588	0.32	10.99	21.85	47.90	49.59*	2.69*
		(0.29-0.34)	(10.29-11.73)	(20.01-23.88)	(43.08-53.25)	(45.70-53.84)	(2.44-2.97)
≥24	163	0.36	10.94	22.85	38.34	61.44*	3.32*
		(0.31-0.42)	(9.67-12.39)	(19.32-27.03)	(31.37-46.85)	(52.56-71.81)	(2.76-4.00)
Hypertension							
No	690	0.32	11.15	21.86	46.73	51.62	2.83
		(0.30-0.35)	(10.50-11.83)	(20.15-23.71)	(42.39-51.52)	(47.85-55.70)	(2.59-3.10)
Yes	61	0.35	9.22	24.52	34.97	55.81	2.65
		(0.27-0.46)	(7.53-11.29)	(18.63-32.27)	(25.18-48.57)	(43.21-72.10)	(1.96-3.60)
DM							

No	735	0.33	11.10	22.15	46.11	52.19	2.84
		(0.30-0.35)	(10.48-11.76)	(20.47-23.97)	(41.93-50.70)	(48.47-56.15)	(2.61-3.10)
Yes	16	0.28	6.48	18.43	28.45	42.00	1.82
		(0.17-0.47)	(4.37-9.60)	(10.77-31.53)	(14.97-54.05)	(25.51-69.20)	(1.01-3.30)
LDL-C (mg/dL)							
<130	626	0.33	11.14	22.15	47.19	51.94	2.86
		(0.30-0.35)	(10.45-11.87)	(20.33-24.14)	(42.61-52.30)	(47.94-56.26)	(2.60-3.15)
≥130	125	0.31	10.20	21.64	38.63	51.98	2.60
		(0.26-0.38)	(8.86-11.74)	(17.95-26.21)	(30.69-48.57)	(43.47-62.12)	(2.10-3.22)
Triglyceride (mg/dL)							
<160	715	0.33	11.21**	22.18	46.97**	51.93	2.79
		(0.30-0.35)	(10.58-11.89)	(20.47-24.02)	(42.69-51.68)	(48.18-55.98)	(2.55-3.05)
≥160	36	0.33	7.16**	19.92	25.84**	52.21	3.48
		(0.24-0.46)	(5.51-9.30)	(13.93-28.50)	(16.88-39.57)	(37.41-72.82)	(2.34-5.17)

$^{*}P < 0.05$

$^{**}P < 0.01$

∑MEHP, sum of (MEHP/278)+(MEHHP/294)+(MEOHP/292)

Supplementary Table S3: Basic demographics of the sample subjects including geometric means and their 95% confidence intervals of the urinary 8-OHdG and 8-isoPGF2α

	No.	8-OHdG (μg/L)	8-isoPGF$_{2α}$(μg/L)
Overall	751	2.95 (2.79-3.10)	1.28 (1.20-1.36)
Age			
12-19	217	3.03 (2.75-3.35)	1.05(0.93-1.19)**
20-30	534	2.91 (2.73-3.10)	1.39 (1.28-1.50)**
Gender			
Female	449	2.61 (2.44-2.79)**	1.20 (1.10-1.31)*
Male	302	3.54 (3.26-3.84)**	1.41 (1.27-1.57)*
Household income			
<50000 NT dollars per month	292	3.01 (2.77 -3.28)	1.25 (1.12-1.40)
≥50000 NT dollars per month	458	2.90 (2.71-3.10)	1.30 (1.19-1.41)
Smoking status			
Non-current smoker	622	2.91 (2.74-3.08)	1.24 (1.15-1.33)
< 10 cigarettes/day	47	2.80 (2.26-3.46)	1.54 (1.18-2.01)
10–19 cigarettes/day	48	3.51 (2.85-4.33)	1.50 (1.15-1.95)
≥ 20 cigarettes/day	34	3.17(2.47-4.06)	1.44 (1.05-1.97)
Current alcohol consumption			
No	682	2.91 (2.75-3.08)	1.28 (1.20-1.38)
Yes	69	3.31 (2.78-3.95)	1.24 (0.99-1.54)
Body mass index (kg/m^2)			
<24	588	3.01 (2.83-3.19)	1.26 (1.16-1.36)
≥24	163	2.74 (2.44-3.07)	1.37 (1.18-1.58)
Hypertension			
No	690	2.95 (2.79-3.11)	1.27 (1.18-1.36)
Yes	61	2.96 (2.46-2.57)	1.43 (1.13-1.80)
DM			
No	735	2.98 (2.83-3.15)**	1.28 (1.19-1.37)
Yes	16	1.68 (1.17-2.41)**	1.34 (0.85-2.13)
LDL-C (mg/dL)			
<130	626	2.99(2.83-3.17)	1.28(1.19-1.37)
≥130	125	2.72(2.39-3.10)	1.30(1.10-1.53)
Triglyceride (mg/dL)			
<160	715	2.98(2.82-3.15)	1.28(1.20-1.37)
≥160	36	2.35(1.85-2.99)	1.22(0.90-1.66)

$^{*}P < 0.05$

$^{**}P < 0.01$
